# Matrix Metalloproteinase-2-Responsive Peptide-Modified Cleavable PEGylated Liposomes for Paclitaxel Delivery

**DOI:** 10.3390/ph18071042

**Published:** 2025-07-15

**Authors:** Xingyu Zhao, Yinghuan Li

**Affiliations:** School of Pharmaceutical Sciences, Capital Medical University, Beijing 100069, China; m13121367488@126.com

**Keywords:** matrix metalloproteinases, paclitaxel, cleavable PEG, breast cancer cells, molecular docking, GPLGVRG peptide, liposome

## Abstract

**Background/Objectives**: PEGylated liposomes are widely recognized for their biocompatibility and capacity to extend systemic circulation via “stealth” properties. However, the PEG corona often limits tumor penetration and cellular internalization. Targeting matrix metalloproteinase-2 (MMP-2), frequently upregulated in breast cancer stroma, presents an opportunity to enhance tissue-specific drug delivery. In this study, we engineered MMP-2-responsive GPLGVRG peptide-modified cleavable PEGylated liposomes for targeted paclitaxel (PTX) delivery. **Methods**: Molecular docking simulations employed the MMP-2 crystal structure (PDB ID: 7XJO) to assess GPLGVRG peptide binding affinity. A cleavable, enzyme-sensitive peptide-PEG conjugate (Chol-PEG_2K_-GPLGVRG-PEG_5K_) was synthesized via small-molecule liquid-phase synthesis and characterized by ^1^H NMR and MALDI-TOF MS. Liposomes incorporating this conjugate (S-Peps-PEG_5K_) were formulated to evaluate whether MMP-2-mediated peptide degradation triggers detachment of long-chain PEG moieties, thereby enhancing internalization by 4T1 breast cancer cells. Additionally, the effects of tumor microenvironmental pH (~6.5) and MMP-2 concentration on drug release dynamics were investigated. **Results**: Molecular docking revealed robust GPLGVRG-MMP-2 interactions, yielding a binding energy of −7.1 kcal/mol. The peptide formed hydrogen bonds with MMP-2 residues Tyr A:23 and Arg A:53 (bond lengths: 2.4–2.5 Å) and engaged in hydrophobic contacts, confirming MMP-2 as the primary recognition site. Formulations containing 5 mol% Chol-PEG_2K_-GPLGVRG-PEG_5K_ combined with 0.15 µg/mL MMP-2 (S-Peps-PEG_5K_ +MMP) exhibited superior internalization efficiency and significantly reduced clonogenic survival compared to controls. Notably, acidic pH (~6.5) induced MMP-2-mediated cleavage of the GPLGVRG peptide, accelerating S-Peps-PEG_5K_ dissociation and facilitating drug release. **Conclusions**: MMP-2-responsive, cleavable PEGylated liposomes markedly improve PTX accumulation and controlled release at tumor sites by dynamically modulating their stealth properties, offering a promising strategy to enhance chemotherapy efficacy in breast cancer.

## 1. Introduction

Globally, breast cancer ranks as the second most diagnosed cancer among females [1]. Despite recent advances in treatment, chemotherapy agents like paclitaxel (PTX) remain pivotal in improving survival rates for advanced breast cancer patients [2]. PTX exerts its primary mechanism by stabilizing microtubules, inhibiting their depolymerization, and thereby disrupting cell cycle progression—particularly during mitosis. By binding to microtubules and forming stable bundles, PTX interferes with cellular division, ultimately triggering apoptosis [3,4]. This mechanism underpins its efficacy against rapidly proliferating cancer cells. Additionally, PTX exhibits immunomodulatory properties, activating host immune responses and enhancing antitumor activity via cytokine release and T cell stimulation [5,6]. However, challenges such as drug resistance, systemic toxicity, and tumor recurrence persist as major barriers to optimal clinical outcomes, underscoring the need for innovative therapeutic strategies [7,8].

Matrix metalloproteinase-2 (MMP-2) has emerged as a critical target in breast cancer due to its elevated expression in the tumor microenvironment [9]. As a zinc-dependent endopeptidase, MMP-2 degrades the extracellular matrix, facilitating tumor cell migration and invasion [10]. Its marked upregulation in breast tumors compared to normal tissues positions MMP-2 as an ideal candidate for targeted drug delivery [11]. The MMP-2-sensitive peptide GPLGVRG undergoes specific cleavage by this enzyme, enabling its integration into PTX delivery systems for spatiotemporal control over drug release [12]. By incorporating GPLGVRG as a linker, these systems achieve tumor-selective PEG detachment, enhancing cellular uptake and intracellular trafficking of PTX while minimizing off-target toxicity [13]. Strategies employing GPLGVRG-modified liposomes or polymeric carriers illustrate the potential of MMP-2-responsive designs in precision oncology [14].

Polyethylene glycol (PEG) is a cornerstone of modern drug delivery systems, renowned for its biocompatibility and ability to prolong circulation via “stealth” properties [15]. However, PEG’s shielding effect often limits tumor penetration and cellular uptake [16]. To address this, PEG cleavage mechanisms—such as enzyme-, pH-, or temperature-responsive linkers—have been developed [17,18,19]. Enzyme-responsive systems, for instance, exploit tumor-enriched enzymes like MMPs; PEG conjugated to MMP-sensitive peptides, e.g., maleimide-PEG-octa peptide-DOPE and PEG-GPLGVRG-poly(*ε*-carprolactone)-poly(3-guanidinopropyl methacrylamide), shed their protective chains in the tumor microenvironment, thereby exposing drug moieties for enhanced internalization [20,21]. While promising, these systems face challenges: ensuring stability under physiological conditions, optimizing cleavage kinetics across diverse tumors, and validating biocompatibility for clinical translation.

This study pioneered a simplified dual-PEG MMP-2-responsive linker (cholesterol-PEG_2K_-GPLGVRG-PEG_5K_) for PTX-loaded liposomes (i.e., S-Peps-PEG_5K_). Conventional PTX-loaded liposomes (Lip), Lip-containing cholesterol-PEG_2K_ (Lip-PEG_2K_), and Lip-containing cholesterol-PEG_2K_-GPLGVRG (S-Peps) were used as controls. Molecular docking first revealed GPLGVRG’s affinity for MMP-2. The dynamic modulation of stealth properties arises from the engineered liposomes’ dual functionality: exploiting PEG for prolonged blood circulation, followed by MMP-2-triggered cleavage of the GPLGVRG peptide, resulted in selective PEG_5K_ shedding within tumor microenvironments. This design augmented PTX uptake in tumor cells, merging targeted activation with controlled release—a step toward overcoming traditional chemotherapeutic limitations. This mechanism was supported by the increased cellular uptake of liposomes after PEG_5K_ detachment. Clone formation and cytotoxicity assays further substantiated MMP-2-triggered cleavage of the GPLGVRG peptide, leading to PEG_5K_ shedding and enhanced cellular internalization, which collectively inhibited tumor cell proliferation and viability. Lastly, the in vitro drug release study identified the tumor microenvironment’s acidic pH (~6.5) as a key trigger for MMP-2-mediated cleavage of the GPLGVRG peptide. The dose-dependent relationship between MMP-2 concentration and drug release validated the GPLGVRG-MMP-2 affinity predicted by molecular docking.

## 2. Results and Discussion

### 2.1. Molecular Docking of MMP-2 in Computer-Aided Drug Design

**Figure 1:**
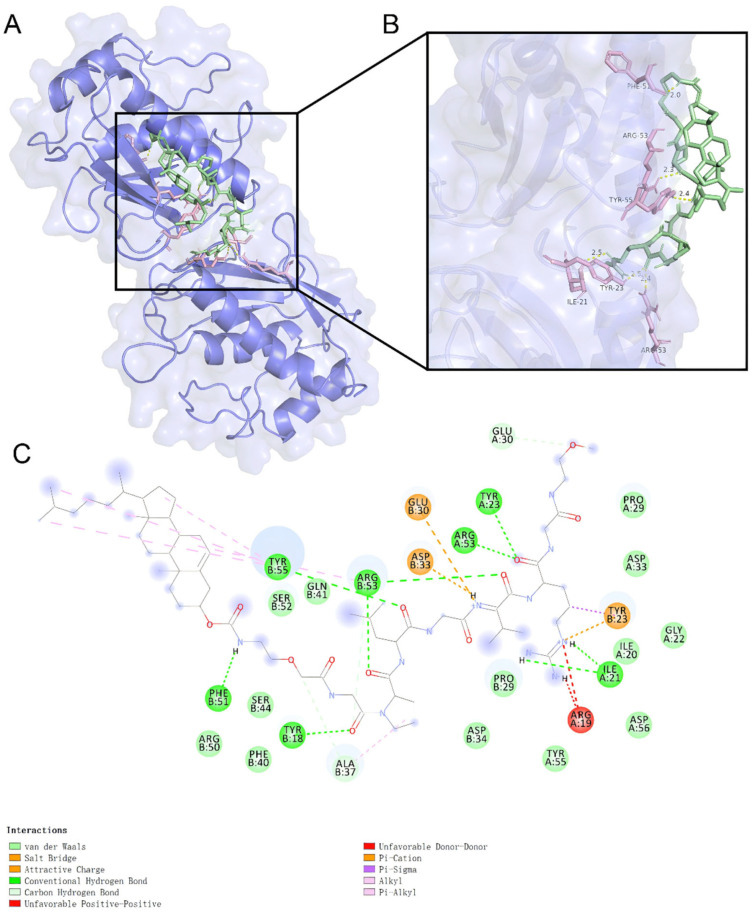
Molecular docking of MMP-2 protein with the ligand GPLGVRG. (**A**) Three-dimensional binding conformation of the ligand within the receptor binding site. (**B**) Magnified view showing key interactions within the binding pocket. (**C**) Two-dimensional interaction diagram between the ligand and receptor, depicting hydrogen bonds, hydrophobic interactions, π–π stacking, and other relevant interactions.

A negative Gibbs free energy change (ΔG) signifies a spontaneous binding interaction between the ligand and receptor [22]. Optimal docking conformations were visualized in both two-dimensional and three-dimensional representations using PyMol 3.1.6.1 and Discovery Studio 2.5.5 (Figure 1A) [23]. Molecular docking simulations, performed with AutoDock Vina 1.2.7, revealed that the peptide ligand GPLGVRG adopted a stable conformation within the MMP-2 binding pocket, forming robust non-covalent interactions with key residues [24]. The calculated ΔG of −7.1 kcal/mol at the binding site underscores strong affinity, with lower binding energies correlating to higher stability.

Detailed interaction analyses (Figure 1B,C) highlighted critical contributions from hydrogen bonds with Tyr A:23 (2.5 Å) and Arg A:53 (2.4 Å), which reinforced binding specificity [25]. Additionally, π–π stacking and hydrophobic effects further stabilized the ligand–receptor complex, illustrating the multifaceted nature of molecular recognition [26]. These findings collectively demonstrate that GPLGVRG exhibits high binding potency for MMP-2, mediated by a synergistic network of non-covalent forces.

### 2.2. Synthesis and Characterization of Chol-PEG_2K_-GPLGVRG and Chol-PEG_2K_-GPLGVRG-PEG_5K_

**Figure 2:**
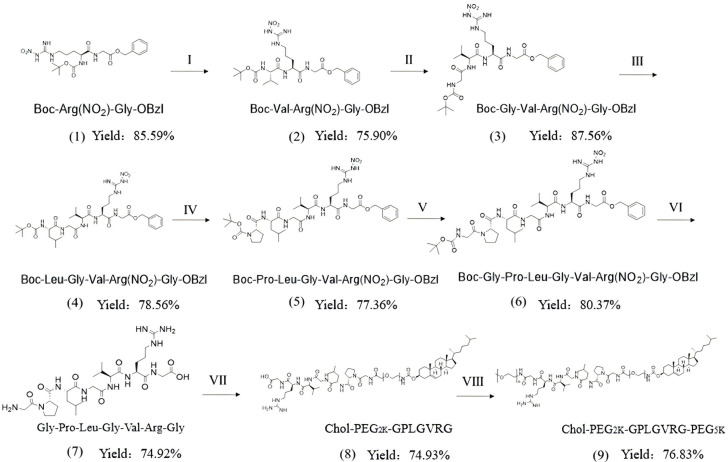
The synthesis route of Chol-PEG_2K_-GPLGVRG and Chol-PEG_2K_-GPLGVRG-PEG_5K_.

**Figure 3:**
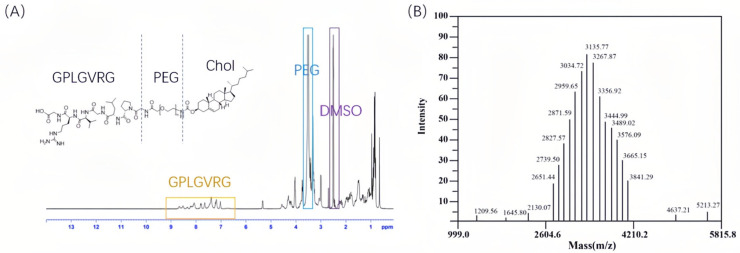
(**A**) ^1^H NMR Spectrum and (**B**) MALDI-TOF MS of Chol-PEG_2K_-GPLGVRG.

**Figure 4:**
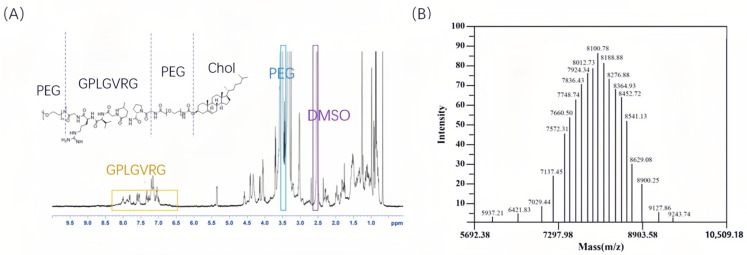
(**A**) ^1^H NMR spectrum and (**B**) MALDI-TOF MS of Chol-PEG_2K_-GPLGVRG-PEG_5K_.

Figure 2 illustrates the chemical synthesis route and yield of Chol-PEG_2K_-GPLGVRG and Chol-PEG_2K_-GPLGVRG-PEG_5K_, while Figure 3 and Figure 4 present the ^1^H NMR spectra and MALDI-TOF MS data for these compounds. In the ^1^H NMR spectrum, the chemical shift for PEG was observed at approximately ~3.5 ppm, whereas the amide bonds in the GPLGVRG peptide sequence exhibited resonances between 7–9 ppm (For detailed data please see Supplementary Materials). Notably, the integrated proton counts for PEG_2K_ and PEG_5K_ were 180 and 450, respectively, corresponding to molecular weights of 3135.71 Da and 8100.78 Da for the PEG moieties in Chol-PEG_2K_-GPLGVRG and Chol-PEG_2K_-GPLGVRG-PEG_5K_, respectively, as confirmed by MALDI-TOF MS [27].

### 2.3. Characterization of MMP-2 Responsive Peptide-Modified Liposomes

Table 1 shows the average particle size, zeta potential, PDI, EE, and DL of various liposomes. The four liposome formulations displayed particle sizes ranging from 100 to 200 nm, which are advantageous for cellular uptake. The PDI values remained below 0.1, confirming highly uniform size distribution. The zeta potential of unmodified Lip was approximately +8.1 mV, while PEGylation introduced a negative surface charge. This shift arises from the polarity and charge properties of the PEG chains: negatively charged functional groups (e.g., carboxyl groups) or conformational effects in the PEG structure can impart an overall negative charge to the liposomes [28]. Consistent with this mechanism, the zeta potential of S-Peps-PEG_5K_ decreased significantly to approximately −26.3 mV.

EE represents the percentage of PTX successfully encapsulated within liposomes relative to the initial PTX amount added. DL capacity denotes the ratio of the total PTX mass to the combined mass of the carrier material and PTX. Due to PTX’s hydrophobic nature, any unencapsulated PTX that fails to integrate into the lipid bilayer is separated from the liposomal suspension via ultrafiltration centrifugation, where it passes through the filter membrane. Across all four PTX-loaded liposome formulations, the EE averaged approximately 90%, with DL capacities ranging from 4% to 5%. Notably, no significant differences in these parameters were observed between groups. Furthermore, all formulations maintained physical stability over 30 days of refrigerated storage at 4 °C, exhibiting negligible alterations in particle size, zeta potential, and EE.

**Figure 5:**
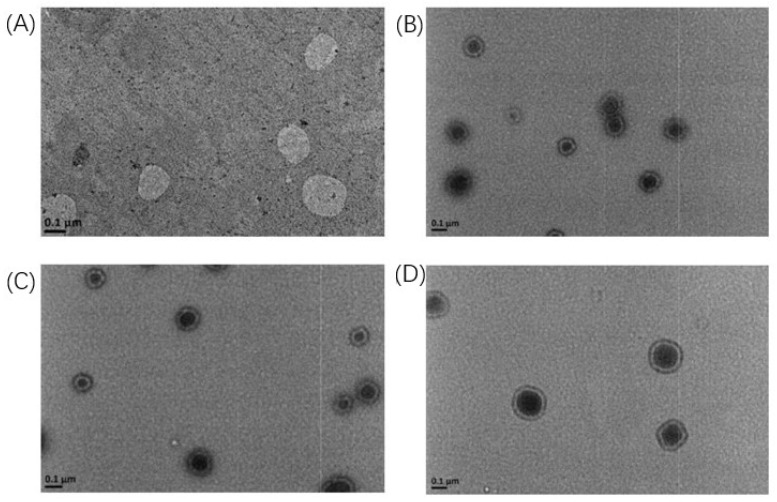
The morphology of liposomes under TEM. (**A**) Lip, (**B**) Lip-PEG_2K_, (**C**) S-Peps, (**D**) S-Peps-PEG_5k_. Scale bar: 100 nm.

Figure 5 depicts the morphology of the four liposomes as revealed by TEM. PEGylated liposomes exhibited distinct dark halo structures, a characteristic feature linked to their composition, structural attributes, and electron scattering properties [29]. These circular or oval halos, corresponding to the lipid bilayer periphery, validated the role of PEG in enhancing hydrophilicity and colloidal stability. The observed halo formation was further attributed to altered electron scattering at the liposome surface due to the presence of PEG chains, which modulated interfacial interactions.

### 2.4. Effects of GPLGVRG and MMP-2 Enzyme on the Quantitative Uptake by 4T1 Cells

**Figure 6:**
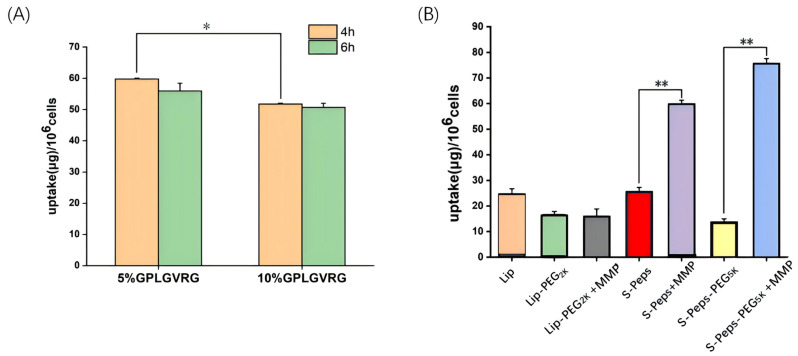
The effects of GPLGVRG and MMP-2 enzyme on the quantitative uptake by 4T1 cells. Cellular uptake of (**A**) S-Peps-PEG_5K_ containing 5 or 10 mol% Chol-PEG_2K_-GPLGVRG-PEG_5K_ combined with MMP (0.15 µg/mL) at 37 °C for 4–6 h and (**B**) various liposomes in 4T1 cells at 37 °C for 4 h (*n* = 4). * *p* < 0.05, ** *p* < 0.01.

Figure 6A reveals comparable cellular uptake at 4 and 6 h, with no significant differences observed. Notably, S-Peps-PEG_5K_ demonstrated superior internalization efficiency when formulated with 5 mol% Chol-PEG_2K_-GPLGVRG-PEG_5K_ compared to 10 mol%, achieving statistical significance (*p* < 0.05).

Figure 6B provides a comparative analysis of cellular uptake across liposome variants. Lip formulations exhibited minimal uptake, while Lip-PEG_2K_ showed a marked reduction in internalization irrespective of MMP-2 activity. After incorporation of the GPLGVRG peptide into Lip-PEG_2K_, S-Peps enhanced uptake, and MMP treatment further doubled uptake efficiency in the S-Peps +MMP group, underscoring the efficacy of enzyme-responsive design in promoting tumor-specific liposome accumulation. In contrast, S-Peps-PEG_5K_ displayed reduced internalization relative to S-Peps, attributed to the extended PEG_5K_ corona, creating a spatial shielding effect. Remarkably, the S-Peps-PEG_5K_ +MMP group exhibited an over fourfold increase in uptake post-MMP treatment. This surge resulted from MMP-2-triggered cleavage of the GPLGVRG peptide, causing PEG_5K_ shedding and subsequent exposure of the liposome surface, thereby facilitating enhanced cellular internalization. These results offer a promising strategy for advancing future in vivo antitumor efficacy studies using 4T1 tumor-bearing mouse models.

### 2.5. Clone Formation Assay for Uptake Efficiency

**Figure 7:**
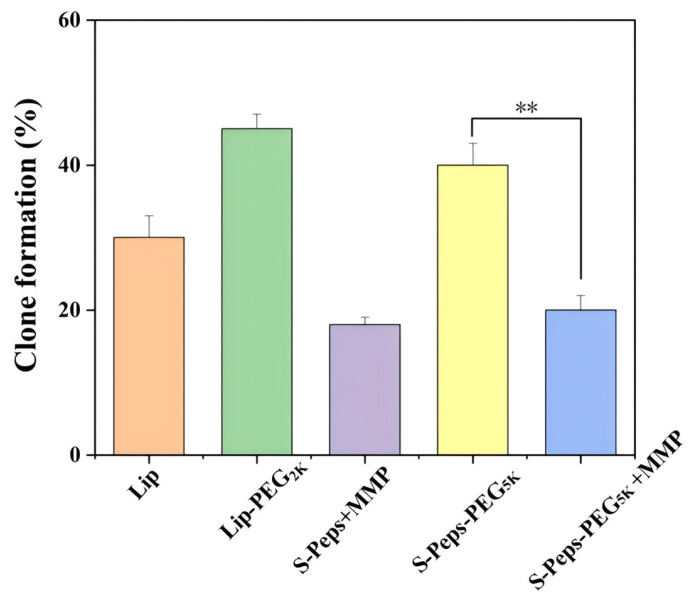
The clonogenic assay for various liposomes. ** *p* < 0.01.

Figure 7 presents the clonogenic assay results for the tested liposome formulations. PEGylated liposomes exhibited elevated clone formation rates due to inefficient cellular uptake, whereas their combination with MMP-2 groups markedly reduced clonogenicity, correlating with enhanced intracellular uptake efficiency. The S-Peps-PEG_5K_ +MMP group exhibited a significantly lower clonogenic rate compared to S-Peps-PEG_5K_ (*p* < 0.01), demonstrating potent suppression of tumor cell proliferation. This effect correlated with enhanced PTX accumulation and cytotoxicity, which disrupted critical cellular proliferation pathways.

### 2.6. Confocal Microscopy for Intracellular Distribution

**Figure 8:**
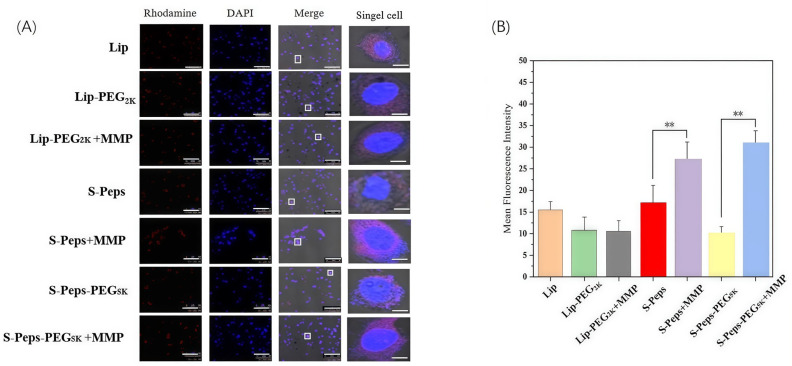
Intracellular distribution of Rhod-modified liposomes in 4T1 cells by confocal microscopy and the calculated mean fluorescence intensity (MFI) using ImageJ 1.8.0 software. (**A**) Confocal pictures (Bar: 50 μm). Single cell in the white box was magnified in the right column (Bar: 10 μm). (**B**) MFI of different formulations (*n* = 4). ** *p* < 0.01.

Confocal microscopy provided concurrent insights into uptake dynamics and subcellular localization (Figure 8). Compared to Lip-PEG_2K_, S-Peps markedly improved cellular uptake efficiency, while MMP-mediated enzymatic cleavage further augmented internalization and intracellular distribution. Notably, S-Peps-PEG_5K_ displayed a 40% reduction in mean fluorescence intensity (MFI) relative to S-Peps (*p* < 0.01), highlighting the masking effect of the extended PEG_5K_ chain on the GPLGVRG peptide. Remarkably, MMP-2 treatment restored MFI in the S-Peps-PEG_5K_ +MMP group to levels comparable with the S-Peps +MMP group, confirming that MMP specifically cleaves the PEG–peptide linkage. This cleavage enables dynamic switching between the “stealth” properties of PEG and the targeting capability of GPLGVRG, optimizing liposome functionality in response to enzymatic triggers.

### 2.7. Cytotoxicity

**Figure 9:**
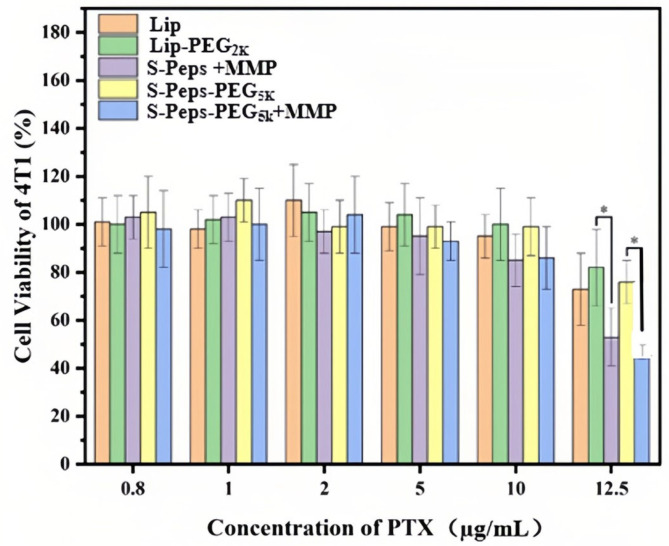
Cell viability of 4T1 cells treated with various liposomes at different concentrations of PTX, combining without or with MMP-2 concentration of 0.15 μg/mL (*n* = 6). * *p* < 0.05.

Figure 9 presents the results of the MTT assay examining 4T1 cell viability following treatment with various liposome formulations. In this assay, a cell survival rate exceeding 80% is typically considered indicative of non-cytotoxic effects. At PTX concentrations below 10 µg/mL, all five liposome formulations tested demonstrated negligible cytotoxicity toward 4T1 cells. At a PTX concentration of 10 µg/mL, both the S-Peps +MMP and S-Peps-PEG_5K_ +MMP groups exhibited marginal reductions in cell viability (*p* > 0.05). However, when the PTX concentration was increased to 12.5 µg/mL, these reductions became statistically significant (*p* < 0.05). This dose-dependent cytotoxicity likely arises from MMP-2-mediated cleavage of the GPLGVRG peptide, enhancing intracellular PTX accumulation and subsequent cytotoxic effects.

**Figure 10:**
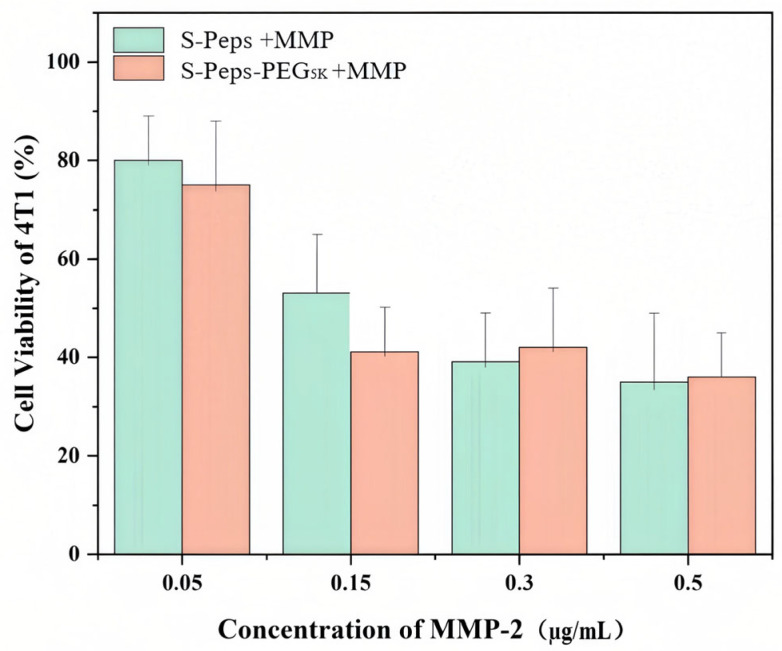
Cell viability of 4T1 cells treated with different concentrations of MMP-2 combined with liposomes at the PTX concentration of 12.5 μg/mL (*n* = 6).

Clinically relevant MMP-2 concentrations at breast cancer lesions (0.15–0.23 µg/mL) were modeled in vitro (Figure 10) [30]. At a fixed PTX concentration of 12.5 µg/mL, where sensitivity to low MMP-2 doses was observed, increasing MMP-2 concentrations from 0.05 to 0.3 µg/mL led to a progressive decrease in cell viability. However, further elevation to 0.5 µg/mL did not elicit additional cytotoxic effects, suggesting a saturation point in MMP-2 responsiveness within this dose range. These findings confirmed that MMP-2-triggered GPLGVRG peptide cleavage exhibited a clear dose-dependent relationship between 0.05 and 0.3 µg/mL MMP-2.

### 2.8. Effects of pH and MMP-2 Enzyme Concentration on Drug Release

**Figure 11:**
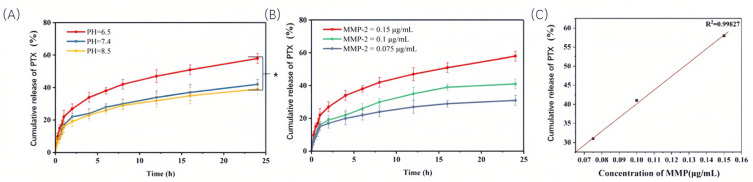
In vitro PTX release experiment (*n* = 4). (**A**) The release curves of S-Peps-PEG_5K_ at different pH values with MMP-2 enzyme concentration of 0.15 µg/mL at 37 °C. (**B**) The release curves of S-Peps-PEG_5K_ at pH 6.5 with various concentrations of MMP-2 at 37 °C, and (**C**) the relationship between cumulative release and the concentration of MMP-2 enzyme. * *p* < 0.05.

The in vitro release profile of S-Peps-PEG_5K_ in PBS at varying pH (6.5, 7.4, and 8.5) with MMP-2 enzyme (0.15 μg/mL) revealed distinct pH-dependent behavior (Figure 11A). Under acidic conditions (pH 6.5), an initial rapid release phase was observed within 4 h, achieving ~35% cumulative release, followed by a gradual increase to ~55% by 24 h. In contrast, neutral (pH 7.4) and alkaline (pH 8.5) conditions significantly suppressed release kinetics, yielding ~35% cumulative release at 24 h (*p* < 0.01 vs. acidic group). These findings underscore the tumor microenvironment’s acidic pH (~6.5) as a critical trigger for MMP-2-mediated cleavage of the GPLGVRG peptide, enabling selective PEG_5K_ shedding within tumors and subsequent enhancement of PTX release from S-Peps-PEG_5K_. Due to its hydrophobic nature, PTX directly penetrated tumor cell membranes without requiring carrier proteins. Local accumulation of liberated PTX created a steep concentration gradient, facilitating passive diffusion into tumor cells. Concurrently, the acidic tumor microenvironment increased cell membrane permeability, thereby amplifying PTX’s passive transmembrane transport. In normal tissues, however, neutral pH and intact blood vessels limited liposome release, mitigating systemic PTX toxicity. Additionally, liposome-encapsulated PTX that persists within liposomes enters cells via endocytic pathways, including lipid raft-, clathrin-, caveolae-, and macropinocytosis-mediated endocytosis [28].

A dose–response relationship between MMP-2 concentration and drug release was further demonstrated at pH 6.5 (Figure 11B). Elevating MMP-2 levels (0.075–0.15 μg/mL) enhanced cumulative release in a linear fashion, underscoring the formulation’s sensitivity to enzymatic activity due to GPLGVRG-MMP-2 affinity within simulated tumor microenvironments. This correlation supported the potential of S-Peps-PEG_5K_ to exploit both low pH and elevated MMP-2 levels characteristic of tumor tissues.

## 3. Materials and Methods

### 3.1. Materials

Boc-Arg(NO_2_), Gly-OBzl·Tos, Boc-Val, Boc-Gly, Boc-Leu, Boc-Pro, Paclitaxel (PTX), 4-aminophenylmercuric acetate (APMA), and collagenase IV (MMP-2) were purchased from Sigma-Aldrich (St. Louis, MO, USA). N,N’-Dicyclohexylcarbodiimide (DCC) was from J&K Scientific (Beijing, China). 1-Hydroxybenzotriazole (HOBt) was obtained from Shanghai Yuanye Bio-Technology Co., Ltd. (Shanghai, China). N-Methylmorpholine (NMM) was from Sinopharm Chemical Reagent Co., Ltd. (Beijing, China). Sodium bicarbonate, sodium chloride, and potassium bisulfate were from Beijing Chemical Works (Beijing, China). Hydrogen chloride (HCl), trifluoroacetic acid (TFA), trifluoromethanesulfonic acid (TfOH), dichloromethane (DCM), petroleum ether (PE), methanol (MeOH), ethyl acetate (EA), and tetrahydrofuran (THF) were purchased from Beijing Institute of Chemical Reagents Co., Ltd. (Beijing, China). Cholesterol (Chol), 1,2-dioleoyl-sn-glycero-3-phosphoethanol-amine-N-(lissamine rhodamine B sulfonyl) ammonium salt (Rhod), and Egg yolk phosphatidylcholine (EPC) were obtained from Avanti (Alabaster, AL, USA). Dimethylformamide (DMF), 1-(3-Dimethylaminopropyl)-3-ethylcarbodiimide hydrochloride (EDC), 4-(Dimethylamino)-pyridine (DMAP), and Triethylamine (TEA) were from Thermo Fisher Scientific (Waltham, MA, USA). Chol-PEG_2K_-NHS and mPEG_5K_-NH_2_ were purchased from Xi’an Qiyue Biotechnology Co., Ltd. (Xi’an, China). (4,5-dimethylthiazol-2-yl)-2,5-diphenyl tetrazolium bromide (MTT), 4′,6-Diamidino-2-phenylindole (DAPI), 4% paraformaldehyde, and Triton X-100 were obtained from Beijing Solarbio Science & Technology Co., Ltd. (Beijing, China). Tris(hydroxymethyl)aminomethane Hydrochloride (Tris-HCl) and phosphate-buffered saline (PBS) were from Gibco BRL (Carlsbad, CA, USA). All the reagents and solvents were analytical or HPLC grade. All materials were used as received.

### 3.2. Molecular Docking of MMP-2 in Computer-Aided Drug Design

Molecular docking analysis was performed to assess interactions between the target protein and small-molecule ligands [31]. The protein’s three-dimensional crystal structure (PDB ID: 7XJO) was retrieved from the Protein Data Bank (PDB). Preprocessing of the structural data, carried out using PyMol 3.1.6.1 molecular visualization software, entailed removing solvent molecules, adding hydrogen atoms, and optimizing the structure. Conformational analysis, which included identifying rotatable bonds, yielded a standardized PDB file [32]. Docking simulations were executed via Autodock 1.2.7 software, with binding free energy (ΔG) serving as the metric for interaction strength. The conformation exhibiting the lowest (most negative) ΔG was designated as the optimal binding pose, furnishing a robust model for later experimental validation.

### 3.3. Synthesis and Characterization of Chol-PEG_2K_-GPLGVRG-PEG_5K_

Chol-PEG_2K_-GPLGVRG-PEG_5K_ was synthesized according to a published method [33]. Briefly, Boc-Arg(NO_2_) (2.00 g, 6.26 mmol), DCC (1.55 g, 7.52 mmol), and HOBt (1.01 g, 7.52 mmol) were dissolved in THF (20 mL) and activated in an ice bath for 30 min. Gly-OBzl·Tos (2.53 g, 7.52 mmol) was added into NMM (2.5 mL) to adjust the pH to 9. Then, the two solutions were mixed and stirred at room temperature for 18 h. After the reaction was completed, the organic solvent was removed under reduced pressure and the extraction was repeated with sodium bicarbonate, sodium chloride, and potassium bisulfate. The organic layers were combined and purified by column chromatography (PE/EA = 6:1) to obtain Boc-Arg(NO_2_)-Gly-OBzl. A solution of HCl-EA (4 N, 17 mL) was added to Boc-Arg(NO_2_)-Gly-OBzl (2.50 g, 5.36 mmol) dropwise at 0 °C for 8 h. The reaction solution was concentrated under reduced pressure, and the residue was repeatedly dissolved by anhydrous EA (30 mL) and dried 4 times to obtain HCl·Arg(NO_2_)-Gly-OBzl. Following the same procedure, Boc-Val (1.63 g, 7.52 mmol) was substituted for Boc-Arg(NO_2_) and reacted with HCl·Arg(NO_2_)-Gly-OBzl (2.50 g, 5.36 mmol), affording Boc-Val-Arg(NO_2_)-Gly-OBzl and HCl·Val-Arg(NO_2_)-Gly-OBzl in turn. Boc-Gly (1.64 g, 7.52 mmol) reacted with HCl·Val-Arg(NO_2_)-Gly-OBzl (2.32 g, 4.37 mmol) to obtain HCl·Gly-Val-Arg(NO_2_)-Gly-OBzl. Boc-Leu (2.34 g, 7.52 mmol) reacted with HCl·Gly-Val-Arg(NO_2_)-Gly-OBzl (2.91 g, 4.56 mmol) to obtain HCl·Leu-Gly-Val-Arg(NO_2_)-Gly-OBzl. Boc-Pro (2.02 g, 7.52 mmol) reacted with HCl·Leu-Gly-Val-Arg(NO_2_)-Gly-OBzl (3.98 g, 4.56 mmol) to obtain HCl·Pro-Leu-Gly-Val-Arg(NO_2_)-Gly-OBzl. Boc-Gly (1.64 g, 7.52 mmol) reacted with HCl·Pro-Leu-Gly-Val-Arg(NO_2_)-Gly-OBzl (4.58 g, 4.56 mmol) to obtain Boc-Gly-Pro-Leu-Gly-Val-Arg(NO_2_)-Gly-OBzl.

Boc-Gly-Pro-Leu-Gly-Val-Arg(NO_2_)-Gly-OBzl (3.54 g, 3.98 mmol) was dissolved in DCM (30 mL), with the addition of 4:1 TFA-TfOH (0.45 mL) solution dropwise at 0 °C for 4 h and sodium bicarbonate to adjust the pH to 7. The organic solvent was removed under reduced pressure. The organic layers were combined and purified by column chromatography (DCM/MeOH = 6:1) to obtain Gly-Pro-Leu-Gly-Val-Arg-Gly (GPLGVRG). Finally, Chol-PEG_2K_-NHS (1.15 g, 5 mmol) was dissolved in DMF (15 mL), while GPLGVRG (1.35 g, 5.5 mmol) was added into TEA (3 mL). The two solutions were mixed and stirred at room temperature for 12 h. The reaction solution was transferred to a dialysis bag (MW = 1000), dialyzing in pure water for 24 h, and then the dialysate was collected via freeze drying to obtain Chol-PEG_2K_-GPLGVRG. Subsequently, Chol-PEG_2K_-GPLGVRG (12.00 g, 5 mmol), EDC (1.15 g, 5 mmol), mPEG_5K_-NH_2_ (7.35 g, 5 mmol), and DMAP (2.32 g, 5 mmol) were again dissolved in DMF (20 mL) and stirred at room temperature for 4 h. The reaction solution was dialyzed in pure water for 24 h and freeze dried to obtain Chol-PEG_2K_-GPLGVRG-PEG_5K_. The structure of Chol-PEG_2K_-GPLGVRG and Chol-PEG_2K_-GPLGVRG-PEG_5K_ were confirmed by ^1^H NMR (Advance 300 MHz NMR, Bruker, Germany) and Matrix-assisted laser desorption/ionization time-of-flight mass spectrometry (MALDI-TOF MS, AB Sciex, Inc., Boston, MA, USA) [34].

### 3.4. Preparation and Characterization of MMP-2-Responsive Peptide-Modified Liposomes

Four groups of liposomes were prepared, including Lip, Lip-PEG_2k_, S-Peps, and S-Peps-PEG_5k_ (Table 2) [28]. Briefly, the raw materials were dissolved in dichloromethane and mixed in a round-bottom flask. The mixture was dried into a thin film at 150 rpm using a rotary evaporator with a stepwise reduction in pressure, followed by lying in a desiccator overnight to remove residual organic solvents. Subsequently, the film was added with ultrapure water containing 10% sucrose and sonicated for 5 min. Hydration was performed in the rotary evaporator at 100 rpm for 1 h, resulting in a coarse liposome suspension, followed by extrusion 19 times through a 100 nm polycarbonate membrane using a liposome extruder (Avanti Polar Lipids, Inc. Birmingham, AL, USA) to reduce the particle size of the liposomes. The prepared liposomes were stored at 4 °C.

The average particle size, zeta potential, and polydispersity index (PDI) of the various liposomes were measured using a 90 Plus PALS Zeta and particle size analyzer (Malvern Instruments, Malvern, UK). The morphologies of the liposomes were observed under transmission electron microscopy (TEM) after negative staining (JEM-1400PLUS, JEOL Ltd., Tokyo, Japan). The encapsulation efficiency (EE) and drug loading (DL) capacity of the PTX liposomes were determined using ultrafiltration centrifugation combined with high-performance liquid chromatography (HPLC). The mobile phase consisted of methanol and water in a ratio of 70:30 (*v*:*v*), with a flow rate of 1 mL/min and the detection wavelength at 227 nm [28].

The calculations for EE and DL were performed using the following formulas:

$$\mathrm{DL}\;(\%)=\frac{The\;amount\;of\;paclitaxel\;in\;liposomes}{Total\;weight\;of\;liposomes}\times 100\%$$

$$\mathrm{EE}\;(\%)=\frac{The\;amount\;of\;paclitaxel\;in\;liposomes-Free\;paclitaxel\;content}{The\;amount\;of\;paclitaxel\;in\;liposomes}\times 100\%$$

### 3.5. Cell Culture

The mouse breast cancer 4T1 cells were purchased from GuangZhou Jennio Biotech Co., Ltd. (Guangzhou, China), cultured in RPMI-1640 medium (Keygen Biotech Corp., Ltd., Nanjing, China), supplemented with 20% (*v*/*v*) fetal bovine serum (FBS) and 1% (*v*/*v*) penicillin/streptomycin (Solarbio Life Sciences Co., Ltd., Beijing, China). Cells were maintained at 37 °C in a humidified atmosphere with 5% CO_2_.

### 3.6. Quantitative Analysis of Cellular Uptake

Rhod was incorporated at 1 mol% into various PTX-loaded liposomes to quantitatively assess cellular uptake [35]. 4T1 cells were seeded in six-well plates at a density of 2 × 10^5^ cells/well and incubated for 36 h. Cells were then treated with MMP at 0.15 µg/mL alongside Rhod-labeled PTX liposomes (5 µg/mL) for 4–6 h. Formulations tested included Lip, Lip-PEG_2K_, S-Peps, and S-Peps-PEG_5K_, with S-Peps-PEG_5K_ formulations containing 5 or 10 mol% Chol-PEG_2K_-GPLGVRG-PEG_5K_. Control groups received Rhod-modified liposomes without MMP. Post-incubation, cells were washed thrice with ice-cold PBS and lysed with 0.5% Triton X-100 for 30 min. Fluorescence intensity (FI) of Rhod was measured at 550/575 nm (excitation/emission) using a microplate reader, and FI values were converted to PTX concentrations via a standard curve.

### 3.7. Clone Formation Assay for Uptake Efficiency

The inhibition of 4T1 cell clone formation reflected the difference in cellular uptake efficiency from various liposomes [36]. Briefly, 4T1 cells were seeded in a Petri dish at a density of 1 × 10^5^ cells and cultured for 24 h. Then, the cells were treated with Lip, Lip-PEG_2k_, S-Peps +MMP (the combination of S-Peps and MMP), S-Peps-PEG_5K_, and S-Peps-PEG_5K_ +MMP for 4 h at concentrations of PTX (5 µg/mL) and MMP (0.15 µg/mL). After washing with ice-cold PBS, the cells were cultured for 10 days, fixed with 4% paraformaldehyde for 30 min and stained with 0.5% crystal violet for 30 min; colonies were counted under a microscope. Clone formation rate (%) was calculated as

$$Clone\;Formation\;\left(\%\right)=\frac{Number\;of\;Colonies}{Initial\;Seeding\;Density}\times 100\%$$

### 3.8. Confocal Microscopy for Intracellular Distribution

The intracellular localization of liposomes was analyzed using a confocal microscope (Leica TCS SP5, Wetzlar, Germany). 4T1 cells were seeded in confocal dishes at a density of 2 × 10^5^ cells and incubated for 24 h. Then, the cells were treated with different Rhod-labeled PTX liposomes at a concentration of 5 µg/mL and incubated at 37 °C for 4 h. After medium removal and triple ice-cold PBS washes, cells were fixed with 4% paraformaldehyde for 20 min, stained with 0.6 µg/mL DAPI for 20 min, and washed thrice with ice-cold PBS. The dishes were then sealed with PBS containing 50% glycerol and observed under a fluorescence confocal microscope. DAPI (nuclei) and Rhod (liposomes) were excited/emitted at 401/421 nm and 557/571 nm, respectively.

### 3.9. Cytotoxicity

To compare the cytotoxicity of various liposomes in vitro, 4T1 cells were seeded at a density of 10^4^ cells per well in 96-well plates and incubated overnight. Different liposome formulations containing PTX at concentrations of 0.8, 1, 2, 5, 10, and 12.5 µg/mL were then added (100 µL per well), followed by incubation at 37 °C for 24 h. The experimental group comprised of S-Peps-PEG_5K_ with or without MMP supplementation, whereas S-Peps combined with MMP served as the positive control, and Lip and Lip-PEG_2K_ acted as the negative controls. After incubation, 25 µL of MTT solution (2.5 mg/mL) was added to each well, and the plates were further incubated at 37 °C for 6 h. Supernatants were subsequently removed, and formazan crystals were dissolved in 150 µL of DMSO. Absorbance at 490 nm was measured for all groups. Cell viability percentages were calculated relative to the blank control, and the IC_50_ values were derived accordingly.

To investigate the effect of MMP-2 concentration on systemic cytotoxicity, S-Peps-PEG_5K_ combined with MMP was designated as the experimental group, with S-Peps +MMP as the control. Using the same protocol, 4T1 cells were treated with PTX-loaded liposomes at 5 µg/mL combined with varying MMP-2 concentrations (0.05, 0.15, 0.3, and 0.5 µg/mL) and assessed via MTT assay.

### 3.10. Impact of pH and MMP-2 Enzyme Concentration on Drug Release In Vitro

The release behaviors of S-Peps-PEG_5K_ in PBS at varying pH levels (6.5, 7.4, and 8.5) and MMP-2 concentrations (0.075, 0.1, and 0.15 µg/mL) were characterized via dialysis. The release medium comprised 0.5% (*w*/*v*) sodium dodecyl sulfate (SDS) in PBS to ensure sink conditions. Aliquots of 2.0 mL of S-Peps-PEG_5K_ were transferred into individual dialysis bags with a molecular weight cutoff (MWCO) of 14,000 Da, sealed securely, and positioned at the base of a paddle-type dissolution apparatus. Each dissolution cup (250 mL capacity) contained 50 mL of pre-warmed release medium. The system was maintained at a constant temperature of (37 ± 0.5) °C with continuous agitation at 50 revolutions per minute (rpm). At designated intervals (0.25, 0.5, 0.75, 1, 2, 4, 6, 8, 12, 16, and 24 h), 1.0 mL of the release medium was withdrawn and immediately replaced with an equivalent volume of pre-warmed fresh medium to maintain constant volume. The withdrawn sampled fractions were filtered through 0.45 µm syringe filters, and the cumulative PTX release was quantified using HPLC following the protocol described in Section 3.4.

### 3.11. Statistical Analysis

All results were extracted at least 3 times and recorded as mean ± standard deviation (SD). Statistical analysis was performed with Prism 9.0 software (GraphPad Software 9.0) via an unpaired two-tailed *t*-test and one-way ANOVA with Bonferroni multiple comparisons. Statistical significance was indicated as * *p* < 0.05, ** *p* < 0.01.

## 4. Conclusions

This study explored MMP-2-responsive GPLGVRG peptide-modified liposomes for targeted PTX delivery. A cleavable, enzyme-sensitive peptide–PEG conjugate (Chol-PEG_2K_-GPLGVRG-PEG_5K_) was synthesized via liquid-phase synthesis. The GPLGVRG peptide was designed to enhance PTX liposome accumulation in tumor microenvironments by leveraging MMP-2-mediated peptide degradation, which triggered the detachment of long-chain PEG moieties and facilitated tumor cell internalization of PTX. Molecular docking simulations using the MMP-2 crystal structure (PDB ID: 7XJO) as the receptor revealed a strong binding affinity, with a calculated binding energy of −7.1 kcal/mol for the GPLGVRG peptide. Notably, the ligand formed hydrogen bonds with MMP-2 residues Tyr A:23 and Arg A:53 (bond lengths: 2.4–2.5 Å) and engaged in hydrophobic interactions, confirming MMP-2 as a critical recognition site for this peptide. These findings demonstrated that MMP-2-responsive, cleavable PEGylated liposomes significantly improved PTX accumulation and release at tumor sites by dynamically modulating their stealth properties, offering a promising strategy for advancing PTX delivery in vivo.

## Figures and Tables

**Table 1:** Pharmaceutical characterization of liposomes ($\bar{X}\pm SD$).

Liposomes	Size (nm)	Zeta (mV)	PDI	EE (%)	DL (%)
Lip	136.8 ± 0.3	8.1 ± 0.5	0.057 ± 0.002	91.4 ± 4.0	4.41 ± 0.26
Lip-PEG_2K_	186.5 ± 1.4	−23.6 ± 0.4	0.078 ± 0.003	92.7 ± 3.5	4.17 ± 0.43
S-Peps	158.9 ± 4.2	−25.8 ± 0.9	0.095 ± 0.003	94.6 ± 2.1	4.22 ± 0.41
S-Peps-PEG_5K_	179.9 ± 0.7	−26.3 ± 0.3	0.060 ± 0.002	90.3 ± 4.5	3.99 ± 0.21

**Table 2:** The composition of the varying liposomal formulations.

Liposomes	Raw Materials	Molar Ratio
Lip	EPC:Chol:PTX	45:50:5
Lip-PEG_2k_	EPC:Chol:Chol-PEG_2k_-NHS:PTX	45:45:5:5
S-Peps	EPC:Chol:Chol-PEG_2k_-GPLGVRG:PTX	45:45:5:5
S-Peps-PEG_5k_	EPC:Chol:Chol-PEG_2k_-GPLGVRG-PEG_5k_:PTX	45:45:5:5

## Data Availability

All data needed to support the conclusions in the paper are presented in the manuscript. Additional data related to this paper may be available from the corresponding author upon request.

## Supplementary Materials

The following supporting information can be downloaded at: https://www.mdpi.com/article/10.3390/ph18071042/s1, Chemical shift of ^1^H NMR spectrum for the synthesis route of Chol-PEG_2K_-GPLGVRG and Chol-PEG_2K_-GPLGVRG-PEG_5K_.
