# Supplementary material for: Matrix Metalloproteinase-2-Responsive Peptide-Modified Cleavable PEGylated Liposomes for Paclitaxel Delivery
Source: Pharmaceuticals (Basel). 2025 Jul 15;18(7):1042. doi: 10.3390/ph18071042 (PMC12298826; doi:10.3390/ph18071042)
Supplement: Supplementary file 1 [file pharmaceuticals-18-01042-s001.zip › pharmaceuticals-3724358-supplementary.pdf]

# Matrix Metalloproteinase-2-Responsive Peptide-Modified Cleavable PEGylated Liposomes for Paclitaxel Delivery

**Xingyu Zhao and Yinghuan Li ***

School of Pharmaceutical Sciences, Capital Medical University School, Beijing 100069, China

## *Supplementary Materials:*

### Chemical shift of $^1$H NMR spectrum for the synthesis route of Chol-PEG$_{2K}$-GPLGVRG and Chol-PEG$_{2K}$-GPLGVRG-PEG$_{5K}$

1.Boc-Arg ($NO_2$)-Gly-OBzl
$^1$H NMR (300 MHz, DMSO) δ/ppm = 8.36 (s, 1 H), 8.07 (s, 1 H), 7.69 (s, 1 H), 7.36 (s, 5 H), 5.12 (s, 2 H), 4.23 (m, 3 H), 3.72 (s, 2H), 3.12 (s, 2H), 1.50 (m, 4 H), 1.37(s, 9 H).

2. Boc-Val-Arg ($NO_2$)-Gly-OBzl
$^1$H NMR (300 MHz, CDCl3) δ/ppm = 7.56 (s, 2 H), 7.46 (s, 1 H), 7.35 (s, 5 H), 7.26 (s, 2 H), 5.30 (s, 1 H), 5.14 (s, 2 H), 4.70 (s, 1 H), 4.04 (s, 3 H), 3.34 (s, 1 H), 3.26 (s, 1 H), 2.10 (d, J = 5.7 Hz, 1 H), 1.83 (s, 2 H), 1.68 (s, 2 H), 1.40 (s, 9 H), 0.97 (d, J = 6.8 Hz, 6 H).

3. Boc-Gly-Val-Arg ($NO_2$)-Gly-OBzl
$^1$H NMR (300 MHz, DMSO) δ/ppm = 8.47 (s, 2 H), 7.88 (d, J = 8.0 Hz, 2 H), 7.36 (s, 5 H), 6.80 (d, J = 8.0 Hz, 1 H), 5.12 (s, 2 H), 4.35 (m, 1 H), 3.92 (m, 3H), 3.12 (s, 2H), 1.94(m, 1 H), 1.57 (m, 4 H), 1.37(s, 9 H), 0.82 (m, 6H).

4. Boc-Pro-Leu-Gly-Val-Arg ($NO_2$)-Gly-OBzl
$^1$H NMR (300 MHz, DMSO) δ/ppm = 8.52 (s, 1 H), 8.26 (t, J = 6.6 Hz, 2 H), 7.97(d, J = 8.0 Hz, 1 H), 7.71 (m, 1H), 7.35 (s, 5 H), 5.12 (s, 2 H), 4.24 (m, 4 H), 3.82 (m, 8H), 3.12 (s, 1 H), 1.96 (dd, J 1= 4.2 Hz, J 2= 11.0 Hz , 1 H), 1.70 (m, 11 H), 1.32(s, 9 H), 0.84 (m, 12 H).

5.Boc-Gly-Pro-Leu-Gly-Val-Arg ($NO_2$)-Gly-OBzl
$^1$H NMR (300 MHz, DMSO) δ/ppm = 8.37 (t, 2 H), 8.04 (m, 3 H), 7.95 (t, J = 5.6 Hz ,1 H), 7.69 (s , 5H), 6.75 (s, 1 H), 5.11 (s, 2 H), 4.22 (m, 4 H), 3.78 (m, 6 H), 3.50 (m, 2 H), 3.16 (d, J = 5.8 Hz, 2 H), 1.93 (m, 4 H), 1.88(m, 3 H), 1.64 (m, 5 H), 1.47 (s, 9 H), 0.82 (m, 12 H).

6.Gly-Pro-Leu-Gly-Val-Arg-Gly
$^1$H NMR (300 MHz, CDCl3) δ/ppm = 12.65 (s, 1 H), 8.25 (m, 5 H), 8.00 (t, J = 5.6 Hz, 1 H), 7.75 (d, J = 8.2 Hz, 2 H), 7.24 (s, 3 H), 4.34 (m, 4 H), 3.82 (s, 2 H), 3.76 (d, J = 9.7 Hz, 4 H), 3.50 (m, 4 H), 3.11 (d, J = 5.8 Hz, 1 H), 1.88 (m, 8 H), 1.51(d, J = 6.6 Hz, 3 H), 0.87 (m, 6 H), 0.82 (m, 6 H).

7. Chol-PEG$_{2K}$-GPLGVRG

$^1$H NMR (300 MHz, DMSO) δ/ppm = 8.66 (m, 1 H), 8.14 (m, 3 H), 7.80 (d, J = 8.4 Hz ,1 H), 7.65 (s, 1 H)7.40 (s, 3 H), 7.22 (m, 2 H), 7.03 (s, 1 H), 7.20 (m, 5 H), 5.34 (s, 1 H), 4.56(s, 1 H), 4.34 (m, 3 H), 4.23 (m, 1 H), 4.06 (s, 4 H), 3.70 (t , J = 3.5 Hz ,4 H)3.53 (s, 180 H), 3.44 (m, 4 H) , 3.03 (s, 3 H), 2.61 (s, 1 H), 2.28 (m, 4 H),1.85(m, 12 H), 1.53 (m, 10 H), 1.34 (m, 4 H), 1.16 (m, 4 H), 0.99 (s, 5 H),0.88 (m, 24 H), 0.67 (s, 3H).

8. Chol-PEG$_{2K}$-GPLGVRG-PEG$_{5K}$

$^1$H NMR (300 MHz, DMSO) δ/ppm = 8.00 (s, 1 H), 7.80 (d, 2 H), 7.59 (d, J = 6.0 Hz ,2 H), 7.33 (d, J = 6.0 Hz , 1H), 7.23 (s, 1 H), 7.20 (m, 5 H), 7.04 (m, 2 H),5.36 (s, 1 H), 4.58 (t , J = 3.9 Hz ,2 H), 4.42 ( d, J = 3.9 Hz ,2 H ), 4.33 (s, 4 H), 4.14 ( d, J = 5.3 Hz ,1 H ), 4.06 (s, 3 H), 3.70 (t , J = 3.5 Hz ,4 H)3.53 (s, 630 H), 3.45 (m, 4 H) , 3.04 (m, 3 H), 2.69 (s, 1 H), 2.35 (m, 2 H), 2.28 (m, 1 H),1.97(m, 2 H), 1.77(m, 5 H), 1.52 (m, 10 H), 1.36 (m, 6 H), 1.26 (s, 7 H), 1.23 (s, 1 H), 1.13 (m, 7 H), 1.01 (m, 2 H),0.99 (s, 4 H),0.92(d, J = 4.8 Hz, 3 H),0.87(dd, J 1= 1.2 Hz , J 2= 5.0 Hz ,9 H), 0.81 (m, 4 H), 0.67 (s, 3H).
